# Insecticide resistance status of *Anopheles arabiensis* in irrigated and non-irrigated areas in western Kenya

**DOI:** 10.1186/s13071-021-04833-z

**Published:** 2021-06-26

**Authors:** Pauline Winnie Orondo, Steven G. Nyanjom, Harrysone Atieli, John Githure, Benyl M. Ondeto, Kevin O. Ochwedo, Collince J. Omondi, James W. Kazura, Ming-Chieh Lee, Guofa Zhou, Daibin Zhong, Andrew K. Githeko, Guiyun Yan

**Affiliations:** 1 Department of Biochemistry, Jomo Kenyatta University of Agriculture and Technology, Nairobi, Kenya; 2 International Center of Excellence for Malaria Research, Tom Mboya University College of Maseno University, Homa Bay, Kenya; 3 School of Public Health and Community Development, Maseno University, Kisumu, Kenya; 4 Center for Global Health & Diseases, School of Medicine, Case Western Reserve University, Cleveland, OH USA; 5 Program in Public Health, College of Health Sciences, University of California at Irvine, Irvine, CA USA; 6 Centre for Global Health Research, Kenya Medical Research Institute, Kisumu, Kenya

**Keywords:** Malaria vectors, Insecticide resistance, Knockdown resistance, Agriculture

## Abstract

**Background:**

Malaria control in Kenya is based on case management and vector control using long-lasting insecticidal nets (LLINs) and indoor residual spraying (IRS). However, the development of insecticide resistance compromises the effectiveness of insecticide-based vector control programs. The use of pesticides for agricultural purposes has been implicated as one of the sources driving the selection of resistance. The current study was undertaken to assess the status and mechanism of insecticide resistance in malaria vectors in irrigated and non-irrigated areas with varying agrochemical use in western Kenya.

**Methods:**

The study was carried out in 2018–2019 in Homa Bay County, western Kenya. The bioassay was performed on adults reared from larvae collected from irrigated and non-irrigated fields in order to assess the susceptibility of malaria vectors to different classes of insecticides following the standard WHO guidelines. Characterization of knockdown resistance (*kdr*) and acetylcholinesterase-inhibiting enzyme/angiotensin-converting enzyme (*Ace-1*) mutations within *Anopheles gambiae* s.l. species was performed using the polymerase chain reaction (PCR) method. To determine the agricultural and public health insecticide usage pattern, a questionnaire was administered to farmers, households, and veterinary officers in the study area.

**Results:**

*Anopheles arabiensis* was the predominant species in the irrigated (100%, *n* = 154) area and the dominant species in the non-irrigated areas (97.5%, *n* = 162), the rest being *An. gambiae* sensu stricto. In 2018, *Anopheles arabiensis* in the irrigated region were susceptible to all insecticides tested, while in the non-irrigated region reduced mortality was observed (84%) against deltamethrin. In 2019, phenotypic mortality was decreased (97.8–84% to 83.3–78.2%). In contrast, high mortality from malathion (100%), DDT (98.98%), and piperonyl butoxide (PBO)-deltamethrin (100%) was observed. Molecular analysis of the vectors from the irrigated and non-irrigated areas revealed low levels of leucine-serine/phenylalanine substitution at position 1014 (L1014S/L1014F), with mutation frequencies of 1–16%, and low-frequency mutation in the *Ace-1*R gene (0.7%). In addition to very high coverage of LLINs impregnated with pyrethroids and IRS with organophosphate insecticides, pyrethroids were the predominant chemical class of pesticides used for crop and animal protection.

**Conclusion:**

*Anopheles arabiensis* from irrigated areas showed increased phenotypic resistance, and the intensive use of pesticides for crop protection in this region may have contributed to the selection of resistance genes observed. The susceptibility of these malaria vectors to organophosphates and PBO synergists in pyrethroids offers a promising future for IRS and insecticide-treated net-based vector control interventions. These findings emphasize the need for integrated vector control strategies, with particular attention to agricultural practices to mitigate mosquito resistance to insecticides.

**Graphic abstract:**

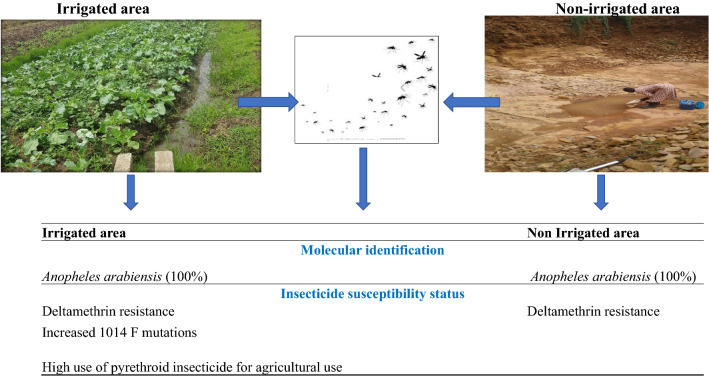

**Supplementary Information:**

The online version contains supplementary material available at 10.1186/s13071-021-04833-z.

## Background

Frequent and prolonged droughts have resulted in the need to increase food production by irrigation in Kenya. This has led to altered ecosystems, with adverse consequences on human health due to water-related diseases [1, 2]. The ongoing deforestation, reclamation of marshlands, and establishment of irrigation systems for food production has resulted in increased vector populations and hence potential malaria transmission risk [3–8]. Agricultural activities have been intensified in many regions in Africa, with increasing interest in household-owned irrigation [9, 10] practices. These agricultural practices have contributed to major health problems such as increased malaria and schistosomiasis transmission [11–17]. Some of the agrochemicals used for crop protection contain the same active chemical ingredients as the insecticides used for public health purposes [18]. One of the unintended consequences of the use of the pesticides for agricultural purposes is the increasing selection for resistance on malaria vectors, as some pesticides used could contaminate nearby mosquito breeding habitats [19–21].

In Kenya, 80% of the land surface area is classified as arid and semi-arid [22, 23], thus necessitating irrigation for food production to sustain the ever-growing population. This has led to the introduction of several irrigation systems across the country, including the Ahero area in western Kenya [24–26] and the Mwea area in central Kenya [14–17], predominantly for rice plantations. *Anopheles arabiensis* has been observed as a predominant malaria vector in arid and semi-arid areas in Kenya [27, 28], and in high-density irrigated areas [14, 29, 30]. This species is an important vector in rice irrigation schemes [31, 32], and exhibits exophilic and significant exophagic behavior [33], increasing its transmission potential, especially outdoors [30, 34, 35]. Although this species shows strong zoophilic behavior, it is partly anthropophilic, depending on the availability of other animal blood meal sources [30, 36, 37]. This vector usually has low sporozoite rates [30, 37]; nevertheless, its high density in irrigated areas makes it an important and probably the major malaria vector in these irrigated areas [14, 30] in the absence of *Anopheles funestus.*

Insecticide resistance has been observed to manifest via two major mechanisms, an increase in metabolic detoxification of insecticides and target-site resistance as a result of mutations. Metabolic detoxification is achieved through the overproduction of cytochrome P450 [38], esterase [39], and glutathione *S*-transferase [40] enzymes in the presence of insecticides. Target-site resistance results from point mutation of genes encoding target proteins that interact with insecticides [41]. These often affect the sodium channels (responsible for pyrethoid and DDT insensitivity), resulting in knockdown resistance (*kdr*), acetylcholine neurotransmitters (hydrolyzed by acetylcholinesterase [AChE] enzyme which is responsible for organophosphate and carbamate insensitivity), and γ-aminobutyric acid (GABA) receptors for cyclodiene and fipronil insecticides. This mutation has been reported in several regions in sub-Saharan Africa [42–46]. In Kenya, the major target-site resistance encountered is the *kdr* [47, 48]. The use of pesticides/insecticides in agriculture and public health has been implicated in the development of insecticide resistance in major malaria vectors [20]. The scale-up of long-lasting insecticide-treated nets (LLINs) and indoor residual spraying (IRS) has been considered the major cause of malaria vector insecticide resistance in many malaria-endemic African countries [49–52]. Extensive use of pyrethroid-based pesticides in agriculture for crop and livestock protection may further enhance/induce resistance [53–55].

The current study was undertaken in an irrigated and non-irrigated areas of Homa Bay County, western Kenya, where malaria vector control with high coverage of pyrethroid-treated LLINs and IRS using pirimiphos-methyl (Actellic^®^300 CS), an organophosphate insecticide, is ongoing [56]. This study aimed to assess the susceptibility status of malaria vectors and the underlying mechanism of resistance in different ecosystems with various agricultural activities. This information will guide mitigation and improved insecticide resistance management strategies.

## Methods

### Study site and design

**Fig. 1:**
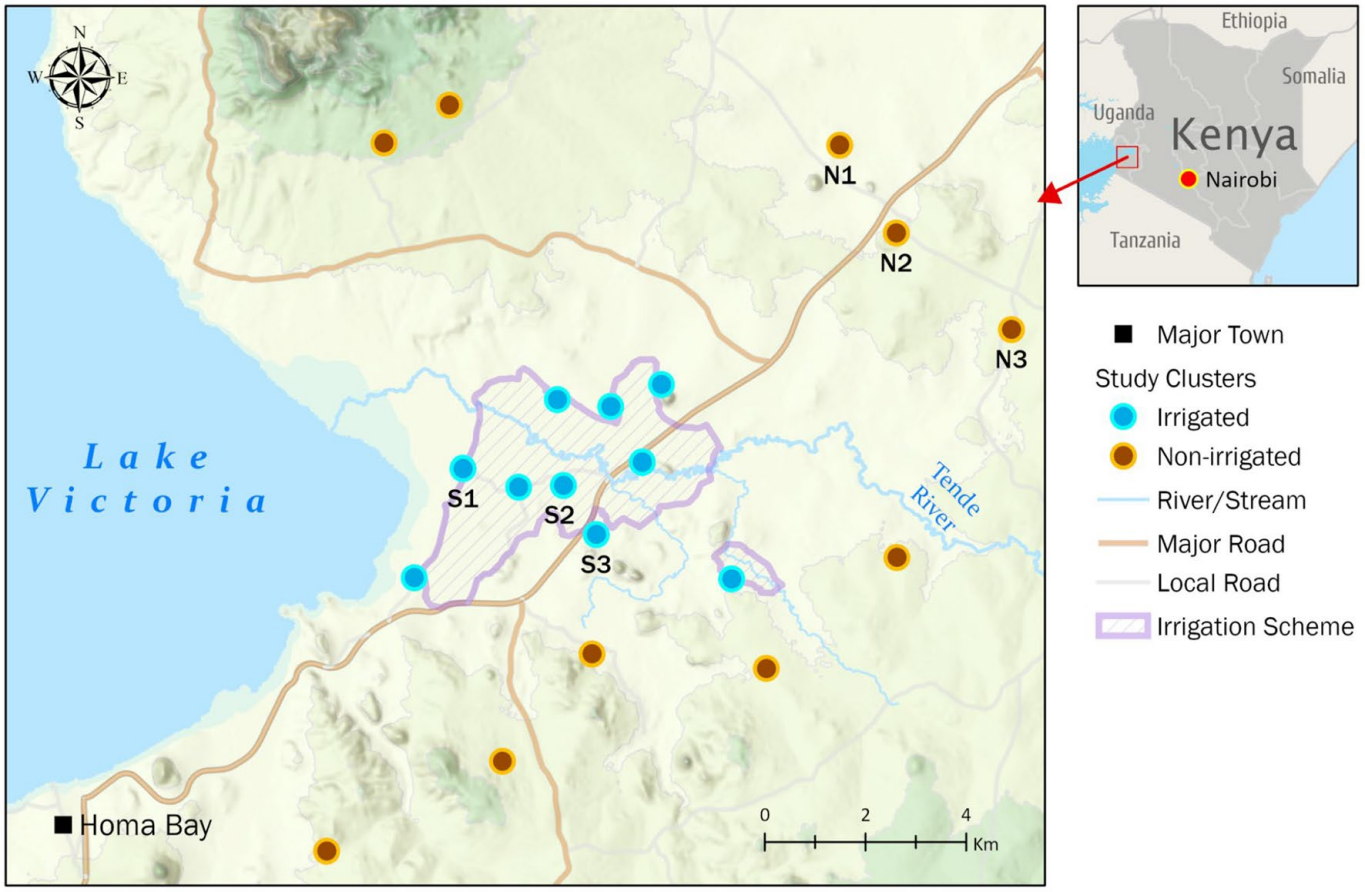
Map of Kenya (right) and Homa Bay study site (in expanded view). Samples were collected from all the above indicated irrigated and non-irrigated areas. S1–S3 and N1–N3 indicate where the questionnaire survey was conducted in the irrigated and non-irrigated areas, respectively

This study was conducted in Homa Bay County, western Kenya, a semi-arid malaria-endemic area situated along the southern shores of Lake Victoria’s Winam Gulf at an altitude of 1040–1330 m above sea level (Fig. 1). This region experiences average annual temperatures of 22.5 °C and rainfall of 1100 mm, with two rainy seasons. The long rains occur between March and May, and the short rains between September and November. A concrete channel irrigation system was constructed in the study area by the Ministry of Environment, Natural Resources and Regional Development Authorities of Kenya in 2007, known as the Kimira-Oluch Small-holder Farm Improvement Project (KOSFIP) (www.afdb.org). This project was undertaken to support subsistence and cash crop production such as cotton and fruits. The local community practice crop and animal farming in addition to fishing. The main malaria vectors are *An. arabiensis* and *An. funestus* sensu lato. Over time, malaria control in this area has relied on pyrethroid-based insecticide-treated nets. However, in 2018 and 2019, IRS was implemented using an organophosphate, pirimiphos-methyl (Actellic^®^ 300 CS), resulting in a dramatic reduction of malaria vector populations [56]. A significant decrease in *An. funestus* s.l. population occurred, tending to near extinction levels [56].

Mosquito larval samples were collected from different village clusters in the irrigated and non-irrigated study areas (Fig. 1). The non-irrigated clusters are at least 2 km from the irrigation channels. Larvae were collected between February and July 2018 and 2019 from all the selected clusters in the irrigated and non-irrigated (10 clusters each) areas (Fig. 1). Various habitat types were sampled during each sampling season. After the long rains (May–July), more habitat types were encountered compared to the dry season (February–March). The habitat types included man-made ponds, swamps, irrigation lining, drainage ditches, natural ponds, river edges, and hoof/footprints.

### Malaria vector larval sampling

Larval sampling was carried out using standard larval 350 ml dippers. Anopheline larvae were collected and transported to the International Center of Excellence for Malaria Research (ICEMR) insectary in Tom Mboya University College, for rearing to adults pending phenotypic insecticide resistance tests and molecular analyses.

### Larval and adult mosquito rearing in the insectary

Field-collected mosquito larvae were placed in larval trays in the larval rearing room and fed a daily diet of Whiskas^®^ cat food (Mars, Incorporated, McLean, VA, USA). Temperature and humidity in the larval room were maintained between 27 and 32°C and 40–60%, respectively. Pupae were collected daily and placed in holding cages covered with mosquito mesh netting where they emerged into adults. Emerged *An. gambiae* s.l. mosquitoes were maintained in the insectary adult rearing room with regulated temperatures (25–28 °C) and humidity (60–75%) and fed 6% glucose solution soaked in cotton wool. The females that emerged in the insectary were used for insecticide resistance bioassays and knockdown resistance (*kdr*) and *Ace-1*enzyme molecular analyses.

### WHO susceptibility test

Two- to five-day-old female adult *An. gambiae* s.l. mosquitoes were used to determine susceptibility to diagnostic concentrations of pyrethroid (0.05% deltamethrin), organophosphate (5% malathion), and organochlorine (4% DDT) insecticides. Standard WHO tube bioassay tests were conducted as per WHO guidelines [57]. Mosquitoes used as control samples were simultaneously placed in WHO tubes lined with untreated papers. Mosquitoes were exposed to insecticide-impregnated test papers for 60 min and the number of knocked down females recorded every 10 min. Fifteen to 20 female mosquitoes were used in each test. At the end of the exposure period, mosquitoes were transferred into holding tubes, maintained on 6% glucose and observed after 24 h for mortality. The positive controls used were an insectary colony of the susceptible *An. gambiae* s.s. Kisumu strain. The final mortality was recorded 24 h post-exposure. Additionally, a synergist piperonyl butoxide (PBO)-deltamethrin exposure bioassay was carried out for a total of 120 min, with 60 min PBO exposure followed by 60 min deltamethrin exposure, with mortality recorded 24 h post-exposure. Knockdown was recorded every 10 min during the exposure period, and mortality counts were recorded after 24 h exposure to PBO alone and PBO-deltamethrin combined. Both live and dead mosquitoes were preserved individually at −20 °C for molecular species identification and the detection of *kdr* mutations and multiple copies of the AChE enzyme encoded by the *Ace-1* gene.

### *An. gambiae* s.l. DNA extraction and species identification

All field-collected specimens were morphologically identified as *An. gambiae* s.l. using Gillies and De Meillon taxonomic keys [58]. DNA from the whole female body was extracted from a proportion of the bioassayed adult females following the Musapa et al. protocol [59]. The molecular species identification was carried out as described by the Scott et al. [60] and Paskewitz and Collins protocols [61].

### Genotyping of *kdr* and *Ace-1* alleles

DNA samples of *An. gambiae* s.l. mosquitoes were assayed to detect the voltage-gated sodium channel (*vgsc*) L1014S (*kdr*-east) and L1014F (*kdr*-west) mutations and mutations in the *Ace-1*gene. Assays were performed on both live and dead mosquitoes post-bioassay tests using the published protocols [62–64].

### Investigation of households using chemicals for public health, agriculture (farms) and veterinary (livestock) purposes

Different questionnaires were prepared and surveys were conducted with randomly selected farmers, households, and veterinary officers/agricultural extension workers to identify the chemicals used in public health, crops, and animal pest control (Additional files 1, 2, 3, 4, 5). Other methods for personal protection against malaria vector and other biting mosquitoes used by the selected households were also surveyed (Additional file 4). The surveys were carried out by trained data clerks sourced from the villages, and the questions were asked in English, Swahili, or the local language (Dholuo), depending on the respondent’s preferred language. The survey lasted between 10 and 20 min. Information recorded included the role of the respondent in the household, farm, or shop; mosquito prevention methods used in the household; crops grown and animals kept; pests that affected the crops and animals; insecticides used in crop and animal pest control; the application frequency of the identified insecticide; how long the insecticide had been used in that particular farm; excess chemical and empty container disposal; and cleaning of empty containers, in addition to other questions (Additional files 1, 2, 3, 4, 5).

### Data management and analysis

During fieldwork, all larval data were entered into Open Data Kit (ODK) on tablets and then uploaded to the online database. The collection site (irrigated or non-irrigated), habitat type, habitat size, and number of larvae sampled were recorded in the field. This was done to identify the most productive habitats. In the insectary and laboratory, data were recorded on the respective laboratory data forms and later entered in Microsoft Excel spreadsheets, followed by error checks and corrections.

The WHO bioassay knockdown was recorded every 10 min for 1 h and final mortality was recorded at 24 h for all test runs with corresponding negative and positive controls. Abbot’s formula was used to correct percentage mortality in cases where the negative control mortality was between 5 and 20%; experiments where negative control mortality was above 20% were discarded and 95% confidence limits of the adjusted mortality determined. Mortality of 98–100% in the sample population indicated susceptibility to the tested insecticide. Mortality of between 90 and 98% suggested possible resistance, and less than 90% mortality indicated resistance in the tested species [57]. Probit analysis was conducted using PoloPlus version 1 software to determine the 50% knockdown time (KDT_50_).

The allele frequencies of *kdr* L1014 mutations and *Ace-1* G119 mutations were determined on Microsoft Excel. Genepop Hardy Weinberg exact tests were used to determine the differences between the *kdr* alleles in the irrigated and non-irrigated areas of both the dead and alive mosquitoes.

Pesticide use questionnaire data were entered and analyzed in Excel. Chi-square and *t* tests were used to determine the significance of the statistical difference between pesticide use in the irrigated and non-irrigated areas.

## Results

### *An. gambiae* s.l. species identification

A total of 1657 female mosquitoes were tested for susceptibility to deltamethrin, malathion, and DDT in 2018 and 2019 (Table 1). Nine hundred and fifty-nine (959) females were identified to species from irrigated and 698 from non-irrigated areas, and they were all *An. arabiensis*.

**Table 1:** The status of phenotypic resistance of *Anopheles arabiensis* and the estimated time to 50% mortality (KDT_50_) when exposed to insecticides for 60 min in the irrigated and non-irrigated areas in 2018 and 2019

Insecticide	Year	Zone	Sample size	KDT_50_(min)	Adjusted mortality rate (%) (95% CI)
Deltamethrin	2018	Irrigated	324	13.0	97.8 (96.2, 99.4)***
		Non-irrigated	114	20.9	84.0 (77.3, 90.7)
	2019	Irrigated	180	23.2	78.2 (72.2, 84.2)
		Non-irrigated	117	21.5	83.3 (76.5, 91.1)
PBO-Deltamethrin	2019	Irrigated	86	8.2	100 (100, 100)
		Non-irrigated	76	7.6	100 (100, 100)
DDT	2019	Irrigated	107	26.1	99.0 (97.1, 100)
		Non-irrigated	123	24.2	100 (100, 100)
Malathion	2018	Irrigated	104	27.0	100 (100, 100)
		Non-irrigated	158	23.2	100 (100, 100)
	2019	Irrigated	158	18.0	100 (100, 100)
		Non-irrigated	111	21.1	100 (100, 100)

*CI* confidence interval

***Significant difference in mortality between irrigated and non-irrigated areas

### *An. arabiensis* insecticide bioassays

Mortality in the positive control Kisumu strain was 100% in all tests, while the mortality in the negative control (wild-caught mosquitoes which were not exposed to insecticide) ranged between 4.7 and 15.4% in all the tests. Phenotypic resistance to deltamethrin was observed in the non-irrigated areas in 2018, with possible phenotypic resistance in the irrigated areas (Table 1). Significantly higher mortality from deltamethrin exposure was recorded in non-irrigated than in irrigated areas in 2018 (*Z* test; *z*-stat = 5.4, *p* < 0.00001). However, in 2019, phenotypic resistance to deltamethrin was observed in both the irrigated and non-irrigated areas, and the mortality rates were comparable between the two areas (78% in irrigated and 83% in non-irrigated area) (Table 1). Susceptibility to malathion (100%) and DDT (98.98–100%) was recorded in both zones in the study site. All the mosquitoes tested on the PBO-deltamethrin combination were susceptible (100%) in irrigated and non-irrigated areas (Table 1). The KDT_50_ of all the chemicals tested was less than 30 min.

### Frequency of *kdr* and *Ace-1* alleles

A total of 317 mosquitoes [both alive (*n* = 38) and dead (*n* = 279)] were tested for the presence of mutations in the *vgsc* gene. Generally, both *kdr*-east and *kdr*-west mutations were observed in both the irrigated and the non-irrigated areas (Table 2). The mutation frequency was low in all tests, ranging from 1 to 16%. However, regardless of study area, no mutation was detected in the *Ace-1* gene in 2018, and very low mutation frequency (0.7%) was found in the non-irrigated area in 2019. The *kdr* allele and genotype frequencies differed significantly between irrigated and non-irrigated zones (Pearson chi-square = 17.804, *df* = 2, *P* = 0.0001 and Pearson chi-square = 14.848, *df* = 4, *P* = 0.012, respectively). The *kdr* genotype results show significant deviation from Hardy–Weinberg equilibrium (HWE) in non-irrigated zones due to heterozygote deficit (*P* < 0.01), while marginally significant heterozygote deficit was observed in the irrigated zone (*P* = 0.0528). Overall, *kdr* genotype frequencies were not consistent with HWE (chi-square > 36.7, *df* = 4, *P* < 0.0001), indicating that the *kdr* allele has experienced strong selective pressure.

**Table 2:** Allele frequency of *vgsc* and *Ace-1* mutations in *Anopheles arabiensis* in irrigated and non-irrigated areas in western Kenya in 2018 and 2019

Year	Zone	vgsc				ACE-1	
		*N*	L1014	L1014S	L1014F	*N*	G119S
2018	Irrigated	81	0.92	0.01	0.07	73	0
	Non-irrigated	72	0.80	0.16	0.04	60	0
2019	Irrigated	76	0.94	0.03	0.03	55	0
	Non-irrigated	88	0.9	0.05	0.05	75	0.007

*N* sample size

### Public health, agricultural and veterinary chemical use

Among the 200 households surveyed (98 were in the irrigated area and 102 in the non-irrigated area), the proportion of households that used LLINs, IRS and other commercial insecticides was 91.8%, 84.4% and 51% in the irrigated area and 91.2%, 91.2% and 39.2% in the non-irrigated area, respectively (Table 3). There was a higher proportion of households in the non-irrigated area (84.3%) that used pesticides in agriculture and veterinary pest control compared to those in the irrigated area (80.6%). There was however no significant difference in the use of public health (vector control) and agricultural (crops)/veterinary (animals) chemicals between the two zones (*t* test; *df* = 6, *t*-stat = 0.1, *p* = 0.9).

**Table 3:** Frequency of responses to chemical use in public health and agriculture (farms) and veterinary (animals) use in households in irrigated and non-irrigated areas

Category	Use	Irrigated (*n* = 98)	Non-irrigated (*n* = 102)	*P* value
Public health	LLINs	91.8 (89.0–94.6%)	91.2 (88.4–94%)	0.88
	IRS	84.4% (77.1–91.6%)	91.2% (85.7–96.7%)	0.14
	Commercial insecticides	51.0 (41.1–60.9%)	39.2 (29.7–48.7%)	0.09
Agricultural/veterinary	Vet and Agric pesticides	80.6 (76.6–84.6%)	84.3 (80.7–87.9%)	0.49

There was no observable difference when the irrigated (75.5%) and the non-irrigated (83.3%) areas were compared in relation to the combined use of public health and agriculture/veterinary chemicals (*t* test; *df* = 10, *t*-stat = 0.2, *p* = 0.9). However, a significantly higher use of pyrethroids was detected in the irrigated than the non-irrigated area (*Z* test; *Z*-stat = 2.7, *p* = 0.007). Households that confirmed the use of chemicals but did not know the chemicals used (unknown classes) were also significantly higher in the non-irrigated area (*Z* test; *Z*-stat = −3.2, *p* = 0.001) (Table 4). No difference in the duration of chemical use for crop protection and livestock pest control (Table 5) was reported (Additional file 6).

**Table 4:** Proportion of households that use chemicals and the chemical classes used

Chemical class	Irrigated (98)	Non-irrigated (102)	*P* value
Use chemicals	75.5 (67.0, 84.0)	83.3 (76.1, 90.5)	0.17
Pyrethroids	62.2 (52.6, 71.8)	28.2 (19.5, 36.9)	< 0.001
Organophosphates	6.8 (1.8, 11.8)	2.4 (0, 5.4)	0.14
Carbamates	2.7 (0, 5.9)	0	0.09
Other classes	21.6 (13.5, 29.7)	4.7 (0.6, 8.8)	< 0.001
Unknown	16.2 (8.9, 23.5)	67.1 (58, 76.2)	< 0.001
Don’t use chemicals	24.5 (16.0, 33.0)	16.7 (9.5, 23.9)	0.17

**Table 5:** Proportion of farmers using pesticides and the duration since the first use of pesticides in agriculture (crops) and livestock (veterinary) in the irrigated and non-irrigated areas

Site/class	*n*	< 12 months	13–36 months	37–60 months	61–120 months	> 120 months	Don’t know
Irrigated	98						
Pyrethroids	46	2.2 (0–4.4)	15.2 (9.9–20.5)	4.3 (1.3–7.3)	26.1 (19.6–32.6)	45.7 (38.4–53)	6.5 (2.9–10.1)
Organophosphates	5	–	60 (38.1–81.9)	–	20 (2.1–37.9)	20 (2.1–37.9)	–
Carbamates	2	–	–	–	–	100 (100)	–
Non-irrigated	102						
Pyrethroids	24	12.5 (5.7–19.3)	8.3 (2.7–13.9)	4.2 (0.1–8.3)	16.7 (9.1–24.3)	45.8 (35.6–56)	12.5 (5.7–19.3)
Organophosphates		–	50 (14.6–85.4)	–	–	50 (14.6–85.4)	–

## Discussion

This study was carried out to determine the levels of susceptibility of *An. arabiensis* to pyrethroid, organophosphate, and organochloride insecticides in an area where public health vector control was undertaken using pyrethroids in insecticidal nets and organophosphates in IRS. The observations herein highlight the importance of multidisciplinary coordination between relevant ministries, including agriculture, public health and environment (IRM plan Kenya, 2020–2024; unpublished). Deltamethrin resistance was observed in both the irrigated and non-irrigated areas. Both *kdr*-east and *kdr*-west mutations were also observed with no *ACE-1* mutations in both irrigated and non-irrigated areas. Additionally, both areas used chemical-based public health interventions for mosquito biting prevention. However, greater use of pyrethroid agricultural pesticides was observed in the irrigated than the non-irrigated area.

With the scaled-up mass distribution of bed nets, increased insecticide resistance in malaria vectors has been reported across sub-Saharan Africa against different classes of insecticides in use (http://www.irmapper.com). The results from this study show moderate phenotypic resistance of *An. arabiensis* against deltamethrin, a chemical compound used in LLINs (DawaPlus), in irrigated and non-irrigated areas. This is an indication that there is increasing resistance against deltamethrin in *An. Arabiensis* in the area. Similar studies indicating increased resistance against pyrethroids in *An. Arabiensis* have been reported in Kenya [65]. This might be due to the contribution from both public health interventions (LLINs) and agricultural activity. One plausible explanation for similar levels of insecticide resistance across areas experiencing different agrochemical exposure intensity may be the wide coverage of public health vector control activities in the region. Previous studies have observed that agricultural chemicals intensify insecticide resistance in areas where public health interventions already exist, due to increased selection pressure [18, 66]. In addition, it was observed that a majority of crop pesticides were used weekly, while the animal pesticides were applied either monthly or every 3 months. This study did not detect resistance against DDT, an indication that the *An. arabiensis* population in this area currently has no cross-resistance between pyrethroids and organochlorides. This is consistent with other studies conducted in Africa, where no cross-resistance was observed in *An. gambiae* s.l. [67] or *An. funestus* [68] species, in contrast to where DDT was previously used for IRS.

The current study reveals higher frequencies of *kdr* compared to previous studies [69]. With the development of reduced nervous sensitivity at the para-type sodium channel, reduced susceptibility to pyrethroid insecticides has been observed, resulting in knockdown resistance. *Kdr* has evolved separately in *An. gambiae* s.s. and *An. arabiensis* [70–72]. In this study, the *kdr* allele and genotype frequencies were higher in the non-irrigated than the irrigated areas. Lower frequencies of *kdr*-east than *kdr*-west were observed in the irrigated region in the study area. This could be a result of agricultural practices, as it has been observed that the mutation might have originally arisen due to the use of agricultural pesticides [73, 74]. The *kdr* genotypes in non-irrigated areas deviated significantly from Hardy–Weinberg equilibrium due to heterozygote deficit, while minimal heterozygote deficit was observed in the irrigated zone. This is an indication that the *kdr* allele has been under strong selective pressure in this area.

*Ace-1* mutation was observed at a very low frequency in the non-irrigated area. This mutation has been associated with carbamate and organophosphate resistance [75]. A recent study from western Kenya observed this mutation at a low frequency in *An. arabiensis* [65]. Residuals from the pesticides used for agricultural purposes could not be dissociated from the observed resistance, as the organophosphates were found to be commonly used for animal pest control. The presence of this mutation in low frequencies in the non-irrigated area is a matter that needs to be further investigated, as an increase in this mutation may impact the gains achieved so far with the ongoing IRS program in the region.

Among the insecticides widely used for agricultural purposes in Homa Bay, pyrethroids appear to be the most common, followed by organophosphates. This reinforces our assumption that the use of these chemicals could be contributing to resistance in this region. When synergist was added to deltamethrin, a pyrethroid in the bioassay, susceptibility was restored, suggesting that the resistance mechanism to this compound involved metabolic pathways, notably monooxygenase enzymes. With the observed susceptibility of the malaria vectors in Homa Bay to PBO-deltamethrin, the introduction of PBO-impregnated nets in the study area will probably be effective in malaria vector control.

## Conclusion

This study revealed increased phenotypic resistance in the *Anopheles arabiensis* from the irrigated area, and the intensive use of pesticides for crop protection in this region may have contributed to the selection pressure resulting in the resistance genes observed. The study findings show that there is a need for continued monitoring of insecticide resistance status, as insecticide resistance poses a major challenge to malaria vector control programs. Additionally, collaboration between the agriculture, public health and environment sectors will be key to insecticide use and resistance management. However, a key limitation of this study was the absence of a comprehensive analysis to ascertain the actual contribution of agricultural/veterinary pesticides to insecticide resistance in *An. arabiensis* in Homa Bay, western Kenya. Further studies are required to determine the insecticide concentration levels in breeding habitats. Such insecticides would likely originate from agricultural pest control activities.

## Supplementary Information

**Additional file 1. **Questionnaire for Agro-vet shops.

**Additional file 2. **Questionnaire for Farmers (Crops).

**Additional file 3. **Questionnaire for Farmers (Animals).

**Additional file 4. **Questionnaire for Households.

**Additional file 5. **Questionnaire for Veterinary Officers/ Agricultural Extension Officers.

**Additional file 6. **Table S1. Proportion of use of different chemical classes in agriculture (farms) and veterinary (animals) in households in irrigated and non-irrigated areas.

## Data Availability

All data generated or analyzed during this study are included in this published article and its Additional files.

## Abbreviations

*Ace*: Angiotensin-converting enzyme
Ach: Acetylcholine
AChE: Acetylcholinesterase
AS-PCR: Allele-specific polymerase chain reaction
DDT: Dichlorodiphenyltrichloroethane
DNA: Deoxyribonucleic acid
F: Phenylalanine
G: Glycine
ICEMR: International Centers of Excellence for Malaria Research
IRS: Indoor residual spraying
*Kdr*: Knockdown resistance
KOSPIF: Kimira-Oluch Small-holder Farm Improvement Project
L: Leucine
LLINs: Long-lasting insecticide-treated nets
ODK: Open Data Kit
PBO: Piperonyl butoxide
S: Serine
s.l.: Sensu lato
s.s.: Sensu stricto
*vgsc*: Voltage-gated sodium channel
WHO: World Health Organization
