# Supplementary material for: Insecticide resistance status of Anopheles arabiensis in irrigated and non-irrigated areas in western Kenya
Source: Parasit Vectors. 2021 Jun 26;14:335. doi: 10.1186/s13071-021-04833-z (PMC8235622; doi:10.1186/s13071-021-04833-z)
Supplement: Supplementary file 1 — Additional file 1. Questionnaire for Agro-vet shops. [file 13071_2021_4833_MOESM1_ESM.doc]

**Questionnaire for Agro-vet shops**

**Introduction**

This ICEMR project is aimed at identifying the common agricultural pesticides used in the control of pests and diseases in the farms and how these pesticides subsequently affect mosquito immature stages and their contribution to malaria transmission. I would like to request for your voluntary participation and to take part in this survey by responding to a few questions stated in this questionnaire.

**Interviewer: ______________ Date of Interview: _________**

**Name of respondent: ____________ Gender: __________________**

**Shop name: __________________ Shop location: __________________**

1. Function of the respondent in the shop: 1= Owner

2 = Manager

3 = Sales personnel

4 = Others (specify)____________

1. Which agricultural pesticides are sold in this shop

__________________________________________________________________________________________________________________________________________________

__________________________________________________________________________________________________________________________________________________

___________________________________________________________________________________________________________________________________________________________________________________________________________________________

1. Which pesticide is frequently bought to be used in the farms for the following infestations

| **Infestation** | **Pesticide** |
| --- | --- |
| Fungal |  |
| Insects (e.g. Beatles |  |
| Virus |  |
| Worms |  |
| Bacteria |  |

1. Which diseases commonly affect the following crops

| **Crop** | **Disease (Fungal/ Beetles/ Virus/ Worms/ Bacterial)** |
| --- | --- |
| Maize |  |
| Millet |  |
| Vegetables |  |
| Fruits |  |
| Green grams |  |
| Rice |  |
| Beans |  |
| Others |  |

1. Do you advise on the following areas when the pesticides are bought:

- Storage Yes No
- Constitution Yes No
- Usage Yes No
- Disposal Yes No
- Expiration date Yes No

1. Please answer the following

| Pesticide used | Which crop is treated | Supplier | Approximate dosage | Frequency of application | Chemical have been used since |
| --- | --- | --- | --- | --- | --- |
|  |  |  |  |  |  |
|  |  |  |  |  |  |
|  |  |  |  |  |  |
|  |  |  |  |  |  |
|  |  |  |  |  |  |
|  |  |  |  |  |  |
|  |  |  |  |  |  |
