# Supplementary material for: Insecticide resistance status of Anopheles arabiensis in irrigated and non-irrigated areas in western Kenya
Source: Parasit Vectors. 2021 Jun 26;14:335. doi: 10.1186/s13071-021-04833-z (PMC8235622; doi:10.1186/s13071-021-04833-z)
Supplement: Supplementary file 2 — Additional file 2. Questionnaire for Farmers (Crops). [file 13071_2021_4833_MOESM2_ESM.docx]

**Questionnaire for Farmers - Crops**

**Introduction**

This ICEMR project is aimed at identifying the common agricultural pesticides used in the control of pests and diseases in the farms and how these pesticides subsequently affect mosquito immature stages and their contribution to malaria transmission. I would like to request for your voluntary participation and to take part in this survey by responding to a few questions stated in this questionnaire.

**Interviewer: ______________ Date of Interview: ______________**

**Name of respondent: ____________ Gender: __________________**

**Village: __________________**

1. Function of the respondent in the farm: 1= Owner

2 = Manager

3 = Farm worker

4 = Others

1. What do you farm? (Tick appropriately)

| **Crop** | **Response** | **Crop** | **Response** |
| --- | --- | --- | --- |
| Maize |  | Green grams |  |
| Millet |  | Rice |  |
| Vegetables |  | Beans |  |
| Fruits (Watermelon, Bananas, Mangoes) |  | Others (specify) |  |

1. Are your crops affected by pests and diseases? Yes No
2. Which pests / diseases commonly affect your crops

| **Crop** | **Disease (Fungal/ Beetles/ Virus/ Worms/ Bacterial)** |
| --- | --- |
| Maize |  |
| Millet |  |
| Vegetables |  |
| Fruits (Watermelon, Bananas, Mangoes) |  |
| Green grams |  |
| Rice |  |
| Beans |  |
| Others |  |

1. a) Do you use pesticides in your farm? Yes No

b) If so please answer the following

| Pesticide name | Where do you buy it | Form of the pesticide (powder, granules, liquid) | How is the pesticide constituted | Mode of application (Spraying, direct) | Approximate dosage for application | Frequency of application | Crop treated | Duration of pesticide use (<6months; 6-12months; 1-3yrs; 3-5yrs; 5-10yrs; >10yrs) |
| --- | --- | --- | --- | --- | --- | --- | --- | --- |
|  |  |  |  |  |  |  |  |  |
|  |  |  |  |  |  |  |  |  |
|  |  |  |  |  |  |  |  |  |
|  |  |  |  |  |  |  |  |  |
|  |  |  |  |  |  |  |  |  |
|  |  |  |  |  |  |  |  |  |
|  |  |  |  |  |  |  |  |  |
|  |  |  |  |  |  |  |  |  |

1. How do you dispose the excess chemical and the empty containers? _______________
2. Where do you wash or clean the equipments used? _____________________________
3. Do you think the chemical gets into the water (rivers/ lake)? _____________________
4. If you do not use pesticides, how do you control pests and weeds __________________
