# Supplementary material for: Insecticide resistance status of Anopheles arabiensis in irrigated and non-irrigated areas in western Kenya
Source: Parasit Vectors. 2021 Jun 26;14:335. doi: 10.1186/s13071-021-04833-z (PMC8235622; doi:10.1186/s13071-021-04833-z)
Supplement: Supplementary file 3 — Additional file 3. Questionnaire for Farmers (Animals). [file 13071_2021_4833_MOESM3_ESM.docx]

**Questionnaire for Farmers - Animals**

**Introduction**

This ICEMR project is aimed at identifying the common insecticides used in the control of pests and diseases on animals and how these chemicals subsequently affect mosquito immature stages and their contribution to malaria transmission. I would like to request for your voluntary participation and to take part in this survey by responding to a few questions stated in this questionnaire.

**Interviewer: ______________ Date of Interview: ______________**

**Name of respondent: ____________ Gender: __________________**

**Cluster: __________________**

1. Function of the respondent in the farm: 1= Owner

2 = Manager

3 = Farm worker

4 = Others (specify) ___________

1. What livestock do you keep? (Tick appropriately)

| **Animals** | **Response** | **Animals** | **Response** |
| --- | --- | --- | --- |
| Cattle |  | Poultry (chicken, ducks, turkey) |  |
| Goats |  | Cat |  |
| Sheep |  | Dog |  |
| Donkey |  | Others (specify)_______________ |  |

1. Are your animals affected by pests? Yes No
2. Which pests commonly affect your animals

| **Animals** | **Pests (Ticks, Mites, Flees)** |
| --- | --- |
| Cattle |  |
| Goats |  |
| Sheep |  |
| Donkey |  |
| Poultry (Chicken, ducks, turkey) |  |
| Cat |  |
| Dog |  |
| Others |  |

1. a) Do you use insecticides on your animals Yes No

b) If so please answer the following

| Insecticide name | Form of the insecticide (powder, granules, liquid) | How is the insecticide constituted | Where applied (On the animal, In the animal shade) | Approximate dosage for application | Frequency of application | Animal treated | Duration of insecticide usage (<6months; 6-12months; 1-3yrs; 3-5yrs; 5-10yrs; >10yrs) |
| --- | --- | --- | --- | --- | --- | --- | --- |
|  |  |  |  |  |  |  |  |
|  |  |  |  |  |  |  |  |
|  |  |  |  |  |  |  |  |
|  |  |  |  |  |  |  |  |
|  |  |  |  |  |  |  |  |
|  |  |  |  |  |  |  |  |
|  |  |  |  |  |  |  |  |
|  |  |  |  |  |  |  |  |

1. Where do you apply the insecticide
2. In the animal shade
3. Within the compound but outside the animal shade
4. Outside the compound
5. In the cultivated/ farm lands
6. Along the river bed or lake shore
7. Others (specify)_____________________________
8. How do you dispose the excess chemical and the empty containers? _______________
9. Where do you wash or clean the equipments used? _____________________________
10. Do you think the chemical gets into the water (rivers/ lake)? _____________________
11. If you do not use insecticide, how do you control pests __________________
