# Supplementary material for: Insecticide resistance status of Anopheles arabiensis in irrigated and non-irrigated areas in western Kenya
Source: Parasit Vectors. 2021 Jun 26;14:335. doi: 10.1186/s13071-021-04833-z (PMC8235622; doi:10.1186/s13071-021-04833-z)
Supplement: Supplementary file 4 — Additional file 4. Questionnaire for Households. [file 13071_2021_4833_MOESM4_ESM.doc]

**Questionnaire for Households**

**Introduction**

This ICEMR project is aimed at identifying the common insecticide used in mosquito control in the households and how these insecticides subsequently affect mosquito and their contribution to malaria transmission. I would like to request for your voluntary participation and to take part in this survey by responding to a few questions stated in this questionnaire.

**Interviewer: ______________ Date of Interview: ____________**

**Name of respondent: ____________ Gender: __________________**

**Cluster Number: ______________**

1. Function of the respondent: 1= Household head

2=Others (specify) ____________

1. How do you protect yourself from mosquito bites and how often do you use the method

| **Prevention method** | **Frequency of use** |
| --- | --- |
| Mosquito nets |  |
| Insecticide spraying |  |
| Physically killing |  |
| Others (specify) _________ |  |
| None |  |
