# Supplementary material for: Insecticide resistance status of Anopheles arabiensis in irrigated and non-irrigated areas in western Kenya
Source: Parasit Vectors. 2021 Jun 26;14:335. doi: 10.1186/s13071-021-04833-z (PMC8235622; doi:10.1186/s13071-021-04833-z)
Supplement: Supplementary file 5 — Additional file 5. Questionnaire for Veterinary Officers/ Agricultural Extension Officers. [file 13071_2021_4833_MOESM5_ESM.doc]

**Questionnaire for Vets**

**Introduction**

This ICEMR project is aimed at identifying the common insecticides used in the control of arthropods on farm animals and how these chemicals subsequently affect mosquito immature stages and their contribution to malaria transmission. I would like to request for your voluntary participation and to take part in this survey by responding to a few questions stated in this questionnaire.

**Interviewer: ______________ Date of Interview: _________**

**Name of respondent: ____________ Gender: __________________**

**Area of duty (Location): ___________**

1. Are you: 1= Self- employed

2 = Government/ county appointed

3 = Other (Specify) _________________

1. Which animals do you normally treat of arthropods and ecto-nematods

| **Crop** | **Common arthropods/ ecto-nematods** |
| --- | --- |
| Cattle |  |
| Goats |  |
| Sheep |  |
| Donkey |  |
| Poultry (Chicken, Ducks, Turkey) |  |
| Cats |  |
| Dogs |  |
| Others (Specify) |  |

1. Do you: 1= Treat the animals personally

2= Advice on the treatment

3= Both

1. Are the animals: 1= Brought to your clinic for treatment

2= Treated in the farms

3= Both

1. Please answer the following

| Chemical name | Form of the chemical (powder, granules. liquid) | How is the chemical constituted (none, diluted) | Mode of application (spraying, dusting) | Where applied (on animal, in animal shade, injected) | Approximate dosage | Frequency of application | Animal treated | Chemical have been used since |
| --- | --- | --- | --- | --- | --- | --- | --- | --- |
|  |  |  |  |  |  |  |  |  |
|  |  |  |  |  |  |  |  |  |
|  |  |  |  |  |  |  |  |  |
|  |  |  |  |  |  |  |  |  |
|  |  |  |  |  |  |  |  |  |
|  |  |  |  |  |  |  |  |  |
|  |  |  |  |  |  |  |  |  |

1. Where do you apply the insecticide
2. In the animal shade
3. Within the compound but outside the animal shade
4. Outside the compound
5. In the cultivated/ farm lands
6. Along the river bed or lake shore
7. Others (specify)_____________________________
8. How do you dispose the excess chemical and the empty containers? _______________
9. Where do you wash or clean the equipments used? _____________________________
10. Do you think the chemical gets into the water (rivers/ lake)? _____________________
