# Supplementary material for: Insecticide resistance status of Anopheles arabiensis in irrigated and non-irrigated areas in western Kenya
Source: Parasit Vectors. 2021 Jun 26;14:335. doi: 10.1186/s13071-021-04833-z (PMC8235622; doi:10.1186/s13071-021-04833-z)
Supplement: Supplementary file 6 — Additional file 6. Table S1. Proportion of use of different chemical classes in agriculture (farms) and veterinary (animals) in households in irrigated and non-irrigated areas. [file 13071_2021_4833_MOESM6_ESM.doc]

**Additional file 1**

**Table S1:** Proportion of use of different chemical classes in agriculture (farms) and veterinary (animals) in households in irrigated and non irrigated areas.

| **Class** | **Active ingredient** | **Pesticide brand name** | **n** | **Irrigated (95% CI)** | **Non- irrigated (95% CI)** |
| --- | --- | --- | --- | --- | --- |
| Pyrethroid | Betacyfluthrin | Thunder | 50 | 66% (52.9-79.1%) | 34% (20.9- 47.1%) |
|  | Bifenthrin | Profile | 29 | 69% (52.2-85.8%) | 31% (14.2- 47.8%) |
|  | Lambdacyhalothrin | Grizzly | 2 | 100% (100%) | 0% (0%) |
|  | Deltamethrin | Vectocid | 4 | 100% (100%) | 0% (0%) |
|  | Cypermethrin | Dip | 7 | 28.60% (0 – 62.1%) | 71.40% (37.5- 100%) |
| Organophosphate | Malathion | Oshothion | 2 | 100% (100%) | 0% (0%) |
|  | Diazinon | Diazol | 4 | 50% (1-99%) | 50% (1-99%) |
| Carbamate | Carbaryl | Sevin | 2 | 100% (100%) | 0% (0%) |
| Others | Amidine | Norotrax, Triatix | 44 | 86.40% (76.3- 96.5%) | 13.60% (3.5- 23.7%) |
|  | Lufenuron | Match | 3 | 66.70% (13.4- 100%) | 33.30% (0 -86.6%) |
|  | Multi- site inhibitors | Sodium | 1 | 0% (0%) | 100% (100%) |
|  | Metalaxyl/ Mancozeb,  Abamectin | Ridomil,  Romectin | 5 | 100% (100%) | 0% (0%) |
